# Factors Associated With the Continuation of Breastfeeding Beyond 12 Months of Age in Brazilian Families

**DOI:** 10.1111/jpc.70195

**Published:** 2025-09-11

**Authors:** Mirene Brandão de Paula, Melissa Moreira Mansur Clemente, Sabrine Teixeira Ferraz Grunewald

**Affiliations:** ^1^ Federal University of Juiz de Fora Juiz de Fora Brazil

**Keywords:** breast feeding, health education, maternal and child health, weaning

## Abstract

**Aim:**

To measure the prevalence of BF amongst Brazilian children aged 12–24 months, assess associated social determinants and evaluate the impact of maternal knowledge about its benefits.

**Methods:**

A cross‐sectional study was conducted between September and December 2024, using an online questionnaire completed by Brazilian mothers with children aged 12–24 months. Sociodemographic data were collected, including maternal and child age, education level, marital status, ethnicity, household income and employment status. Additional information was gathered on prenatal care, breastfeeding within the first hour of life, guidance received on BF and factors related to the continuation/interruption of BF.

**Results:**

Over 70% of the 403 mothers surveyed were breastfeeding beyond 12 months. The main influencing factors in the continuation of BF were knowledge of its benefits (87.6%) and personal desire (78.4%). Attending more than six prenatal visits, being aware of WHO recommendations and receiving guidance on the maternal benefits of BF and on milk expression/storage were significantly associated with breastfeeding beyond 12 months of age (*p* < 0.05). Having a younger child negatively impacted prolonged BF (*p* = 0.005).

**Conclusions:**

Prolonged BF is influenced by multiple factors, and healthcare professionals can play a key role in promoting it by implementing health education strategies focused on the maternal benefits of breastfeeding and milk expression/storage. Ensuring adequate prenatal care and family planning is also essential.

**Summary**

- What is already known on this topic
  ○ Prolonged breastfeeding brings benefits to both mother and baby.
  ○ Breastfeeding practices are influenced by several cultural, social, economic and geographic factors.
  ○ Few studies have specifically evaluated factors that promote or discourage breastfeeding beyond the baby's first year of life.
- What this paper adds
  ○ Mothers are often given guidance on breastfeeding, but the guidance does not always cover all relevant topics.
  ○ The main factors indicated by mothers as being related to the interruption of breastfeeding before the first year of life were difficulties with breastfeeding, such as latch‐on or cracked nipples, and fatigue or exhaustion with the process.
  ○ Guidance on extracting and storing milk and on the maternal benefits of breastfeeding impacts the prevalence of prolonged breastfeeding, and should therefore be incorporated by health professionals.

## 1 Introduction

The World Health Organization (WHO) and major medical societies advocate exclusive breastfeeding (EBF) for the first 6 months of life. After this period, continued breastfeeding is recommended alongside the introduction of complementary foods for as long as mutually desired by the mother–child dyad, potentially up to 2 years or beyond [1]. Prolonged breastfeeding, defined as breastfeeding beyond the first 6 months up to 24 months or more, has a significant positive impact on maternal health, with well‐documented reductions in the incidence of type 2 diabetes, hypertension, breast cancer and ovarian cancer [1, 2]. Despite these well‐established benefits, the decision to continue breastfeeding for an extended period often lacks adequate support. Mothers may face ridicule and misinformation regarding their choice, and difficulty discussing such matters with healthcare professionals [2].

In 2023, aiming to provide clearer recommendations on the subject, the WHO published updated guidelines on complementary feeding for infants and young children aged 6–23 months. These guidelines endorse breastfeeding beyond 2 years and emphasise the need for a supportive environment and appropriate services to make this goal achievable [3]. The document highlights the lack of workplace accommodations for breastfeeding mothers, including insufficient availability of childcare facilities and breastfeeding‐friendly infrastructure. It also points to the need for robust support and counselling systems, improved regulation against exploitative marketing by formula manufacturers, and the importance of evidence‐based guidance from healthcare providers [3].

Parallel studies identify maternal knowledge of breastfeeding benefits and the absence of younger siblings or a new pregnancy as motivational factors for continued breastfeeding [4]. In 2021, the WHO, in collaboration with the United Nations Children's Fund (UNICEF), released the ‘Indicators for Assessing Infant and Young Child Feeding Practices’, which underscore the importance of continued breastfeeding from 12 to 23 months for optimal child health outcomes [5]. This document highlights the role of breastfeeding in preventing up to half of all infectious disease‐related deaths in this age group, improving performance in intelligence tests administered during childhood and adolescence, and preventing dehydration in illnesses associated with poor appetite, due to the ease of ingesting breast milk compared to solid foods [5].

In the context of breastfeeding in Brazil, according to the indicators proposed by the Global Breastfeeding Collective (GBC), the country has failed to produce the required 5‐yearly breastfeeding rate reports, which limits accurate understanding of the current situation. The GBC, a partnership between WHO, UNICEF, and other major international agencies, reports that 71% of Brazilian mothers continue breastfeeding during the first year of life, with a notable drop to 45% at 2 years of age [6]. Data from the Brazilian Institute of Geography and Statistics (IBGE) from 2020, however, show a 22.7‐fold increase in continued breastfeeding in the first year and a 23.5‐fold increase in children under 2 years of age compared to data from 1986 [7]. Despite these encouraging trends, there is still a lack of national investment in the promotion of prolonged breastfeeding [6].

Despite the well‐established benefits of breastfeeding and international recommendations, both Brazilian and international literature have primarily focused on breastfeeding during the first 6 months of the infant's life. Factors such as maternal education, household income, marital status, sanitation conditions, number of children, access to prenatal care and media exposure appear to influence the prevalence of prolonged breastfeeding [4]. This prevalence also varies widely across countries and continents: for instance, breastfeeding rates at 12 months of age reach up to 91.2% in Central Africa but remain below 30% in Western Europe [8]. However, there is a lack of studies that explore in detail the factors influencing prolonged breastfeeding rates in different countries or that propose strategies to encourage this practice.

The decision to breastfeed and for how long is influenced by cultural, social, economic and health‐related factors. However, there remains a scarcity of quantitative research in the national literature on this subject. Therefore, the present study aims to estimate the prevalence of prolonged breastfeeding amongst Brazilian children aged 12–24 months and to assess the associated social determinants, focusing particularly on whether maternal knowledge of its health benefits for both mother and child is associated with higher rates of prolonged breastfeeding.

## 2 Materials and Methods

This was a cross‐sectional study conducted through an online questionnaire completed by mothers of infants aged 12–24 months, between 1 September and 31 December 2024. Although data collection was carried out online, the study was conducted at the Federal University of Juiz de Fora and received prior approval from the Research Ethics Committee for Human Subjects (approval number 6.633.449, CAAE 75313723.7.0000.5147). All participants provided informed consent before taking part in the study.

The questionnaires were distributed online, primarily through social media platforms and discussion forums focused on pregnancy and motherhood. A pilot study with 20 participants was conducted to assess the clarity and comprehensibility of the questionnaire; however, the data from this phase were not included in the final analysis.

Inclusion criteria were being 18 years of age or older, being the mother of a child aged between 12 and 24 months and residing in Brazil. The sample size was calculated considering a 95% confidence interval and a 5% margin of error. The estimated population was based on the number of live births in Brazil prior to the study period (2 561 922 live births in 2022, according to data from the Brazilian Ministry of Health) [9], resulting in a required sample size of 384 participants. To account for potential sample losses, an additional 10% of participants were included in data collection.

Responses were excluded if obtained from individuals under 18 years of age, residing outside Brazil, with children outside the defined age range, or if the data collection tool was not fully completed within the research timeframe.

Sociodemographic variables collected included maternal age, child's age, educational level, marital status, state of residence, ethnicity, family income and employment status. Additional questions addressed antenatal care, type of delivery, breastfeeding within the first hour of life and guidance received on breastfeeding: timing of the guidance, topics covered, other sources of information beyond healthcare professionals and knowledge of WHO recommendations.

Mothers were asked whether they had breastfed their child at any point during the previous 24 h. This question was used to divide the questionnaire into two pathways: mothers who were still breastfeeding answered questions regarding factors that may have influenced the continuation of prolonged breastfeeding (such as family support, knowledge of breastfeeding benefits, personal motivation, amongst others); mothers who had ceased breastfeeding were asked about potential reasons for discontinuation (such as breastfeeding fatigue, work‐related issues or personal health concerns). The questionnaire used in this study is available in Data S1.

Data collected through the online questionnaires were exported to Microsoft Excel. Statistical analyses were performed using the Statistical Package for the Social Sciences (SPSS), version 22.0. Descriptive analysis included calculations of means, standard deviations and absolute and relative frequencies. The *χ* ^2^ test was used to assess associations between prenatal and delivery‐related variables, as well as breastfeeding guidance provided by healthcare professionals and the continuation of breastfeeding beyond the first year of life. A significance level of 5% was adopted for all statistical tests.

## 3 Results

A total of 425 responses were obtained; however, 22 were excluded (6 from participants residing in other countries and 16 from participants whose children were not within the 12–24‐month age range), resulting in 403 responses analysed. Maternal age ranged from 18 to 49 years, with a mean of 32.9 years. The mean age of the participants' children was 16.4 months. Most participants (91.9%) were married or in a stable union, and 62.8% of the sample had completed higher education. Nearly 80% of participants resided in the Southeast region of Brazil. A total of 67.8% of respondents reported engaging in paid work, either within or outside the home, and most mothers contributed partially to the family income (52.6%). Sociodemographic data are detailed in Table 1.

**TABLE 1:** Sociodemographic data of the sample (*n* = 403).

	Average	Standard deviation
Mother's age (years)	32.9	5.8
Infant age (months)	16.4	3.9
	*n*	*%*
Marital status		
Married or living with a partner	370	91.9
Single	28	6.9
Separated or divorced	4	1.0
Widow	1	0.2
Education		
Incomplete elementary school	4	1.0
Complete elementary	10	2.5
Incomplete medium	4	1.0
Complete medium	83	20.6
Incomplete higher education	49	12.2
Completed higher education	253	62.8
Ethnicity		
White Brazilian	239	58.6
Brown Brazilian	123	30.5
Black Brazilian	40	9.9
Other	4	1.0
Region of residence		
Southeast	315	78.2
South	38	9.4
Northeast	30	7.4
Midwest	13	3.2
North	7	1.7
Family income		
Up to 1 minimum wage	57	14.1
From 1 to 3 minimum wages	101	25.1
From 3 to 5 minimum wages	96	23.8
From 5 to 10 minimum wages	86	21.3
Above 10 minimum wages	63	15.6
Work status		
Works outside the home.	199	49.4
Works from home	74	18.4
Not working	130	32.3
Contribution to family income		
Mother is the main source of income	70	17.4
Mother contributes partially to the income	212	52.6
Does not contribute to income	120	29.8

Most participants received complete prenatal care (95.3%) and gave birth via caesarean section (64.5%). Slightly more than three‐quarters of the mothers were able to breastfeed within the first hour of life. Regarding breastfeeding education, most information was provided by healthcare professionals during pregnancy (51.1%), and additional information was frequently obtained through television, social media and the internet (78.4%). The most frequently discussed topics were the benefits of breastfeeding for the baby (93.8% of mothers received guidance on this topic) and correct latch and positioning (89.3%). Further details on health education aspects are presented in Table 2.

**TABLE 2:** Clinical information on pregnancy and childbirth and health education received by participants (*n* = 403).

	*n*	%
Delivery		
Caesarean section	260	64.5
Spontaneous vaginal	114	28.3
Vaginal induced	29	7.2
Prenatal visits		
6 or more	384	95.3
Less than 6	11	2.7
Don't remember	8	2.0
Twin pregnancy	3	0.7
Breastfeeding in the first hour	312	77.4
When you received information from health professionals about breastfeeding		
During pregnancy	206	51.1
During hospitalisation for childbirth	176	43.7
At baby checkups	195	48.4
In postpartum consultations	105	26.1
Never	54	13.4
Other sources of information about breastfeeding		
Television, social networks, internet	316	78.4
Friends and family	142	35.2
School or college	32	7.9
Others	86	21.3
What topics did you receive information about?		
Benefits of breastfeeding for the baby	378	93.8
Grip and position	360	89.3
Benefits of breastfeeding for the mother	226	56.1
Milk extraction and storage	226	56.1
Donation to human milk bank	149	37.0
Breastfeeding after the first year of life	145	36.0
Weaning	21	5.2
Mothers who still breastfeed	291	72.2
Mothers who know the WHO recommendation	395	98.0
	*Average*	*Standard deviation*
Length of exclusive breastfeeding (months)	5.3	2.2

Most participants (72.2%) were still breastfeeding at the time of the survey, and most (63.9%) intended to continue breastfeeding until their child reached at least 2 years of age. The main factor contributing to the continuation of breastfeeding was knowledge of its benefits (87.6%), followed by personal motivation (78.4%). Amongst mothers who were no longer breastfeeding, approximately one‐third had ceased breastfeeding within the first 6 months of life. The primary reasons cited for discontinuation were breastfeeding difficulties (34.8%) and maternal fatigue (30.4%). These findings are detailed in Table 3.

**TABLE 3:** Factors indicated by mothers as related to the interruption or maintenance of breastfeeding.

	*n*	%
Mothers who maintained breastfeeding (*n* = 291)		
Until when do you intend to continue breastfeeding?		
Until the baby is less than 2 years old	57	19.6
Up to 2 years or more of the baby	186	63.9
No date set	38	13.1
Other or no information	10	3.4
Factors that contribute to continued breastfeeding		
Knowledge about the benefits of breastfeeding	255	87.6
Personal wish	228	78.4
Flexibility at work	106	36.4
Support from health professionals	86	29.6
Support from family and friends	79	27.1
Lower cost	61	20.9
Mothers who no longer breastfeed (*n* = 112)		
Age at which the baby stopped being breastfed		
< 6 months	38	33.9
6 months to 1 year	33	29.5
1–2 years	41	36.6
Factors that contributed to the interruption of breastfeeding		
Difficulties with breastfeeding (such as fissures, incorrect latch)	39	34.8
Fatigue and exhaustion from breastfeeding	34	30.4
Work‐related issues	27	24.1
Maternal and/or family preference	26	23.2
Illness and/or hospitalisation of the baby	12	10.7
Maternal illnesses or use of medications incompatible with breastfeeding	8	7.1
New pregnancy or birth of another child	7	6.3
Lack of support from family/friends	6	5.4

Several factors were significantly associated with continued breastfeeding beyond the first year of life, as shown in Table 4. These included receiving complete prenatal care and knowledge of WHO recommendations regarding breastfeeding duration, as well as receiving specific guidance on the maternal benefits of breastfeeding and on milk expression and storage. Other specific types of guidance, as well as the timing at which the guidance was received, did not show significant associations.

**TABLE 4:** Factors associated with maintaining breastfeeding after the baby's first year of life.

	AM after 1 year (*n* = 291)	No BF after 1 year (*n* = 112)	*χ*2	*p*
Prenatal care				
6 visits or more	282	102	9576	**0.008**
Less than 6 visits	7	4		
Don't remember	2	6		
Delivery				
Caesarean section	192	68	0.981	0.61
Spontaneous vaginal	79	35		
Vaginal induced	20	9		
Have a younger child				
Yes	41	29	7850	**0.005**
No	250	83		
Knowledge of WHO recommendations				
Yes	289	106	9064	**0.003**
No	2	6		
Has a paid work				
Yes	140	59	0.675	0.41
No	151	53		
Main contributor to family income				
Yes	44	26	3691	0.55
No	247	86		
Breastfeeding first hour				
Yes	225	87	0.006	0.94
No	66	25		
*Received information about AM…*				
At pregnancy				
Yes	149	57	0.03	0.96
No	142	55		
At hospitalisation for childbirth				
Yes	125	51	0.219	0.64
No	166	61		
At baby checkups				
Yes	135	60	1669	0.19
No	156	52		
In postpartum visits				
Yes	73	32	0.51	0.48
No	218	80		
*Received information on the topic…*				
Benefits of breastfeeding for the baby				
Yes	271	107	0.806	0.37
No	20	5		
Grip and positioning				
Yes	256	104	2024	0.16
No	35	8		
Benefits of breastfeeding for the mother				
Yes	174	52	5865	**0.02**
No	117	60		
Milk extraction and storage				
Yes	175	51	7001	**0.008**
No	116	61		
Donation to human milk bank				
Yes	106	43	0.134	0.71
No	185	69		
Breastfeeding after 1 year of life				
Yes	112	33	2859	0.09
No	179	79		
Weaning				
Yes	12	9	2506	0.11
No	279	103		

*Note*: Chi‐squared test, significance level: *p* < 0.05.

## 4 Discussion

In this study, over 70% of participating mothers were still breastfeeding their children after 12 months of age. The main factors associated with continued breastfeeding, as reported by the mothers, were knowledge about its benefits and personal motivation. Amongst mothers who had already weaned their children, most did so during the first year of life, primarily due to breastfeeding difficulties and maternal fatigue. Factors significantly associated with breastfeeding beyond 12 months included attending more than six prenatal visits, awareness of WHO recommendations and having received guidance on the maternal benefits of breastfeeding as well as on milk expression and storage.

In the studied sample, most mothers were white and had completed higher education. Socioeconomic status and educational level often influence various aspects related to access to health services. It is possible that families with better socioeconomic conditions have greater access to prenatal and paediatric care, as demonstrated by Brazilian studies on the topic [10, 11]. Although review studies have shown that, in general, ethnic minorities experience less favourable breastfeeding outcomes [12], studies evaluating sociodemographic aspects in Brazil have reported variable findings. A Brazilian study from 2010 found that maternal education level and ethnicity were not associated with breastfeeding prevalence during the first 6 months of life [13]; however, other sociodemographic factors—such as higher household income and living in rural areas—increased the likelihood of breastfeeding in that age group. A cohort study conducted in the Brazilian Northeast showed that lower education levels were associated with early interruption of EBF, but found no differences related to ethnicity [14]. None of these studies, however, explored the impact of sociodemographic factors on breastfeeding beyond the first 6 months of the infant's life.

The caesarean section rate in the present sample was 64.5%. A study analysing data from 2014 to 2020 reported a national caesarean rate of 56.1% in Brazil, with notable variation between public (up to 47.7%) and private (up to 69.4%) healthcare services, depending on factors such as multiparity, gestational age and foetal position [15]. In the present study, the mode of delivery did not influence the prevalence of prolonged breastfeeding. Previous studies also found no association between delivery mode and breastfeeding prevalence during the first 6 months of life in Brazilian children [13, 14], but those studies did not extend their analyses beyond the initial half‐year of the infant's life.

To support milk supply and direct breastfeeding, skin‐to‐skin contact immediately after birth and breastfeeding initiation within the first or second hour of life are recommended [16]. However, in the present study, early breastfeeding was not significantly associated with continued breastfeeding beyond the first year. Previous studies have shown that breastfeeding within the first hour of life is positively associated with EBF at 6 weeks, 3 months and 6 months [17, 18]. Nonetheless, this effect may not be sustained long enough to influence prolonged breastfeeding.

Most studies have assessed the impact of educational interventions on outcomes such as EBF at 6 months. For example, a cross‐sectional study conducted in Primary Health Units in Rio de Janeiro found that mothers who received guidance about EBF until 6 months had a 32% higher prevalence of this practice compared to those who did not receive such guidance [19]. These findings reinforce the crucial role of healthcare professionals, particularly those in primary care, in promoting and supporting breastfeeding through family counselling.

Although fewer studies have focused specifically on prolonged breastfeeding, there is evidence that health education strategies positively affect breastfeeding rates beyond the first year [20]. Additionally, the present study found that specific guidance—such as information on maternal benefits and milk expression/storage—was significantly associated with prolonged breastfeeding, suggesting these topics should be emphasised during prenatal and postpartum education.

In this study, attending more than six prenatal consultations was positively associated with extended breastfeeding. A Brazilian study showed that over 98% of pregnant women receive prenatal care in the country, and approximately 70% attend at least six prenatal visits. However, significant regional disparities exist, with women in the North and Northeast regions of Brazil being at higher risk of initiating prenatal care later and attending fewer than six visits [21]. A 2016 systematic review showed that of seven studies that examined breastfeeding outcomes, six reported higher breastfeeding prevalence in intervention groups exposed to educational strategies during prenatal care. Common interventions included group prenatal care and home visits by healthcare professionals [22]. Moreover, professional training of healthcare workers is essential for breastfeeding promotion and support, as well as for enhancing family engagement with health systems [22].

Similarly, a 2023 study aligned with WHO recommendations evaluated various prenatal breastfeeding education programs and their outcomes in the postpartum period. These programs included family‐centred approaches, individual education methods and group‐based interventions, all aimed at providing practical support and improving breastfeeding adherence. Evaluated outcomes included breastfeeding self‐efficacy, adherence, knowledge and attitudes towards breastfeeding and family support. Overall, the findings suggest that successful breastfeeding practices are intrinsically linked to prenatal education and maternal engagement [23].

Beyond education, several other factors can influence breastfeeding prevalence. In the present study, breastfeeding difficulties and maternal fatigue were the most frequently cited reasons for early weaning. A 2023 Brazilian cohort study found that male infants, early pacifier use (within the first week of life), and less than 3 months of EBF were associated with an increased risk of early weaning before 2 years of age [24]. Another Brazilian study highlighted maternal reports emphasising early challenges in establishing breastfeeding [25], with similar findings on latch difficulties and maternal fatigue being linked to early discontinuation [26].

Another factor associated with a lower likelihood of continued breastfeeding in the present sample was having a younger child at home. This result mirrors findings from a study conducted in Haiti, which evaluated prolonged breastfeeding [4]. These findings suggest the need for discussions about family planning and contraceptive methods to increase birth intervals and ensure longer breastfeeding durations for each child. A study that assessed breastfeeding rates at 1 year of age across multiple countries also showed that these rates vary according to income–being higher in lower‐income countries–and by continent, with higher rates observed in Latin America (64.5%) compared to North America (38.3%) and Western Europe (28.5%) [8]. However, this regional variation may be influenced by several factors, such as income, education level, and cultural and social aspects, which were not explored in that study.

Given the numerous benefits of breastfeeding, some studies suggest that early weaning may result from a lack of maternal knowledge regarding the quality and importance of breast milk for infant development [27]. Public policies play a key role in strengthening breastfeeding practices by implementing strategic educational programmes and mass media campaigns that disseminate accurate information and counter misinformation commonly found on social media [27].

The recommendations of the Brazilian Ministry of Health are similar to those of the World Health Organization, advocating EBF up to 6 months of age and continued breastfeeding until at least 2 years. These recommendations are compiled in a national dietary guideline for children up to 2 years of age, which provides comprehensive information on breastfeeding, including topics such as milk expression and storage and the maternal health benefits of breastfeeding [28]. However, a study analysing the impact of Brazilian public policies promoting breastfeeding revealed that significant challenges remain, such as the lack of formal implementation across all municipalities, excessive workloads for primary care professionals, and insufficient monitoring of outcomes [29].

This study has limitations, including a predominantly Southeast Brazilian sample, with high educational attainment, mostly white women, and those married or in stable relationships. These characteristics may limit the generalisability of findings to the broader and more diverse Brazilian population. Additionally, due to the cross‐sectional design, causal relationships cannot be established, nor can the sample be followed over time. Moreover, the online distribution of the survey may have favoured the selection of participants with greater access to the internet. The absence of multivariate analysis also weakens the associations found. Nonetheless, the study's strengths include its large sample size with representation from all Brazilian regions and the collection of specific data on breastfeeding‐related health education provided to participants. Its originality is also noteworthy, given the scarcity of Brazilian studies specifically evaluating prolonged breastfeeding.

Given the wide range of cultural, economic, and health‐related factors influencing breastfeeding decisions, research in this area is essential to guide strategies that reduce early weaning. This study highlighted the importance of complete prenatal care, maternal knowledge of breastfeeding guidelines, and specific education on the maternal benefits of breastfeeding and milk expression and storage. Healthcare professionals should incorporate these findings into their counselling practices to support prolonged breastfeeding.

It is also important to highlight the need for further research exploring aspects of prolonged breastfeeding, as the literature predominantly focuses on breastfeeding during the first 6 months of life. Moreover, longitudinal studies are needed to assess factors impacting this practice. Finally, intervention studies are necessary to evaluate strategies that effectively promote breastfeeding, including the maintenance of the practice beyond the infant's first year of life.

## Ethics Statement

This study was conducted at the Federal University of Juiz de Fora and received prior approval from the Research Ethics Committee for Human Subjects (approval number 6.633.449, CAAE 75313723.7.0000.5147).

## Conflicts of Interest

The authors declare no conflicts of interest.

## Supporting information


**Data S1:** Supporting Information.

## Data Availability

The data that support the findings of this study are available on request from the corresponding author. The data are not publicly available due to privacy or ethical restrictions.

## References

[1] American College of Obstetricians and Gynecologists (ACOG) , “ACOG Committee Opinion No. 756: Optimizing Support for Breastfeeding as Part of Obstetric Practice,” Obstetrics and Gynecology 132, no. 4 (2018): e187–e196, 10.1097/AOG.0000000000002890. 30247365

[2] J. Y. Meek and L. Noble , “Section on Breastfeeding. Policy Statement: Breastfeeding and the Use of Human Milk,” Pediatrics 150, no. 1 (2022): e2022057988, 10.1542/peds.2022-057988. 35921640

[3] World Health Organization , WHO Guideline for Complementary Feeding of Infants and Young Children 6–23 Months of Age (World Health Organization, 2023), https://iris.who.int/bitstream/handle/10665/373358/9789240081864‐eng.pdf. 37871145

[4] S. Decelles , M. Nardocci , A. Mildon , et al., “Determinants of Continued Breastfeeding in Children Aged 12‐23 Months in Three Regions of Haiti,” Revista Panamericana de Salud Pública 8, no. 48 (2024): e6, 10.26633/RPSP.2024.6. PMC10921905 38464872

[5] World Health Organization , Ageing and Health: A Policy Brief (World Health Organization, 2020), https://www.who.int/publications/i/item/9789240018389.

[6] D. S. Melo , M. H. de Oliveira , and D. d. S. Pereira , “Brazil's Progress in Protecting, Promoting and Supporting Breastfeeding From the Perspective of the Global Breastfeeding Collective,” Revista Paulista de Pediatria 39 (2021): e2019296, 10.1590/1984-0462/2021/39/2019296. 32876303 PMC7457464

[7] Federal University of Rio de Janeiro , “Infant Feeding I: Prevalence of Feeding Indicators for Children Under 5 Years,” ENANI 2019—Electronic Document (UFRJ, 2021), https://enani.nutricao.ufrj.br/download/relatorio‐4‐aleitamento‐materno/.

[8] P. A. R. Neves , J. S. Vaz , F. S. Maia , et al., “Rates and Time Trends in the Consumption of Breastmilk, Formula, and Animal Milk by Children Younger Than 2 Years From 2000 to 2019: Analysis of 113 Countries,” Lancet Child Adolesc Health 5, no. 9 (2021): 619–630, 10.1016/S2352-4642(21)00163-2. 34245677 PMC8376656

[9] Ministry of Health—Brazil , “Live Birth Information System,” 2024, http://plataforma.saude.gov.br/natalidade/nascidos‐vivos/.

[10] J. S. Saavedra , J. A. Cesar , and A. O. Linhares , “Prenatal Care in Southern Brazil: Coverage, Trend and Disparities,” Revista de Saúde Pública 53 (2019): 40, 10.11606/S1518-8787.2019053000968. 31066818 PMC6542475

[11] A. M. A. Santos , J. Perelman , P. A. Jacinto , et al., “Income‐Related Inequality and Inequity in Children's Health Care: A Longitudinal Analysis Using Data From Brazil,” Social Science & Medicine 224 (2019): 127–137, 10.1016/j.socscimed.2019.01.040. 30772611 PMC6411923

[12] K. M. Jones , M. L. Power , J. T. Queenan , and J. Schulkin , “Racial and Ethnic Disparities in Breastfeeding,” Breastfeeding Medicine 10, no. 4 (2015): 186–196, 10.1089/bfm.2014.0152. 25831234 PMC4410446

[13] D. Wenzel , R. Ocaña‐Riola , G. Maroto‐Navarro , and S. B. de Souza , “A Multilevel Model for the Study of Breastfeeding Determinants in Brazil,” Maternal & Child Nutrition 6, no. 4 (2010): 318–327, 10.1111/j.1740-8709.2009.00206.x. 21050386 PMC6860883

[14] T. O. Vieira , G. O. Vieira , N. F. de Oliveira , C. M. Mendes , E. R. Giugliani , and L. R. Silva , “Duration of Exclusive Breastfeeding in a Brazilian Population: New Determinants in a Cohort Study,” BMC Pregnancy and Childbirth 14 (2014): 175, 10.1186/1471-2393-14-175. 24885939 PMC4046501

[15] V. B. Pereira , S. N. dos Reis , F. G. Araújo , T. Amorim , E. F. Martins , and M. S. Felisbino‐Mendes , “Trends in Cesarean Section Rates in Brazil by Robson Classification Group, 2014‐2020,” Revista Brasileira de Enfermagem 77, no. 3 (2024): e20230099, 10.1590/0034-7167-2023-0099. 39082532 PMC11290736

[16] UpToDate , “Breastfeeding: Parental Education and Support,” cited March 22, 2025, https://www.uptodate.com/contents/breastfeeding‐parental‐education‐and‐support.

[17] A. Prian‐Gaudiano , D. Horta‐Carpinteyro , and A. Sarmiento‐Aguilar , “Relationship Between Skin‐To‐Skin Contact During the First Hour of Life and Duration of Exclusive Breastfeeding,” Boletín Médico del Hospital Infantil de México 81, no. 1 (2024): 10–15, 10.24875/BMHIM.23000160. 38503328

[18] V. Raghavan , B. Bharti , P. Kumar , K. Mukhopadhyay , and L. Dhaliwal , “First Hour Initiation of Breastfeeding and Exclusive Breastfeeding at Six Weeks: Prevalence and Predictors in a Tertiary Care Setting,” Indian Journal of Pediatrics 81, no. 8 (2014): 743–750, 10.1007/s12098-013-1200-y. 24113879

[19] J. d. S. Alves , M. I. C. d. Oliveira , and R. V. V. F. Rito , “Guidance on Breastfeeding in Primary Health Care and the Association With Exclusive Breastfeeding,” Ciênc saúde Coletiva 23, no. 4 (2018): 1077–1088. 10.1590/1413-81232018234.10752016 29694586

[20] J. Zhang , L. Shi , D. F. Chen , J. Wang , and Y. Wang , “Effectiveness of an Educational Intervention to Improve Child Feeding Practices and Growth in Rural China: Updated Results at 18 Months of Age,” Maternal & Child Nutrition 9, no. 1 (2013): 118–129, 10.1111/j.1740-8709.2012.00447.x. 23020102 PMC6860631

[21] M. do Carmo Leal , A. P. Esteves‐Pereira , E. F. Viellas , R. M. S. M. Domingues , and S. G. N. da Gama , “Prenatal Care in the Brazilian Public Health Services,” Revista de Saúde Pública 54 (2020): 8, 10.11606/s1518-8787.2020054001458. 31967277 PMC6961968

[22] E. P. da Silva , R. T. de Lima , and M. M. Osório , “Impact of Educational Strategies in Low‐Risk Prenatal Care: Systematic Review of Randomized Clinical Trials,” Ciência & Saúde Coletiva 21, no. 9 (2016): 2935–2948. 27653079 10.1590/1413-81232015219.01602015

[23] J. Kehinde , C. O'donnell , and A. Grealish , “The Effectiveness of Prenatal Breastfeeding Education on Breastfeeding Uptake Postpartum: A Systematic Review,” Midwifery 118 (2022): 103579. 36580847 10.1016/j.midw.2022.103579

[24] P. S. Mosquera , B. H. Lourenço , A. Matijasevich , M. C. Castro , and M. A. Cardoso , “Prevalence and Predictors of Breastfeeding in the MINA‐Brazil Cohort,” Revista de Saúde Pública 57, no. 2 (2023): 1–13. 38422331 10.11606/s1518-8787.2023057005563 PMC10897961

[25] N. H. Fazzioni and K. Lerner , “Agencies of Women Who Breastfeed: Reflecting on Breastfeeding, Motherhood, and the Internet in Brazil,” Interface 28 (2024): e220698.

[26] J. C. Kent , E. Ashton , C. M. Hardwick , et al., “Nipple Pain in Breastfeeding Mothers: Incidence, Causes and Treatments,” International Journal of Environmental Research and Public Health 12, no. 10 (2015): 12247–12263, 10.3390/ijerph121012247. 26426034 PMC4626966

[27] E. A. Silva , O. T. A. Leite Freitas , C. Barbosa Spinelli , et al., “Health Educational Practices of Exclusive Breastfeeding: A Study in a Neonatal ICU,” Brazilian Journal of Implantology and Health Sciences 5, no. 3 (2023): 575–608.

[28] Ministério da Saúde, Secretaria de Atenção Primaria à Saúde, Departamento de Promoção da Saúde , Guia alimentar para crianças brasileiras menores de 2 anos (Ministério da Saúde, Secretaria de Atenção Primaria à Saúde, Departamento de Promoção da Saúde, 2019), https://www.gov.br/saude/pt‐br/composicao/saps/promocao‐da‐saude/guias‐alimentares/publicacoes/guia_da_crianca_2019.pdf.

[29] D. Melo , S. Venancio , and G. Buccini , “Brazilian Strategy for Breastfeeding and Complementary Feeding Promotion: A Program Impact Pathway Analysis,” International Journal of Environmental Research and Public Health 19, no. 16 (2022): 9839, 10.3390/ijerph19169839. 36011476 PMC9408563

