# Supplementary material for: Factors Associated With the Continuation of Breastfeeding Beyond 12 Months of Age in Brazilian Families
Source: J Paediatr Child Health. 2025 Sep 11;61(12):1842–9. doi: 10.1111/jpc.70195 (PMC12689486; doi:10.1111/jpc.70195)
Supplement: Supplementary file 1 — Data S1: Supporting Information. [file JPC-61-1842-s001.docx]

**Supplementary Material**

**Questionnaire**

Part 1: Sociodemographic data

1. How old are you (in years)?

2. How old is your child at the moment, in years and months?

3. How would you define your ethnicity? Do you consider yourself:

( ) White ( ) Black ( ) Brown ( ) Yellow/Oriental ( ) Indigenous ( ) other__________

4. In which Brazilian state do you live?

5. What is your highest level of education?

a) Incomplete elementary education

b) Complete elementary education

c) Incomplete high school education

d) Complete high school education

e) Incomplete higher education

f) Complete higher education

6. Are you currently pregnant?

a) Yes b) No

7. Besides your child aged between 12 and 24 months, do you have any other younger children?

a) Yes b) No

8. Average family income:

9. Do you have paid work?

a) Yes, I work outside the home

b) Yes, I work from my own home

c) No

10. To what extent does your monthly income contribute to your family's expenses?

a) My job is my family's main source of income.

b) My work contributes partially to my family's income

c) I do not contribute financially to my family's expenses

Part 2: Breastfeeding Information

Now we're going to ask you some questions about pregnancy, birth, and breastfeeding for your child who is between 12 and 24 months old.

11. Your child was born in a birth:

a) Spontaneous vaginal

b) Vaginal after induction

c) Caesarean section

12. How many prenatal appointments did you have during your child's pregnancy?

a) Less than 6 consultations

b) 6 or more consultations

c) I don't remember

13. After birth, was your child breastfed in the first hour of life?

a) yes

b) No

14. When did you receive information about breastfeeding from healthcare professionals (you can select more than one)?

a) During pregnancy

b) During hospitalization for the birth of the baby

c) In the baby's consultations after birth

d) In the mother's consultations after childbirth

e) I did not receive information about breastfeeding at any of these times.

15. In addition to the information provided by health professionals, how else have you learned about breastfeeding (you can select more than one option)?

a) Through television, social networks, internet searches

b) Through friends or family

c) I learned about the topic at school/college

d) Other

16. What topics related to breastfeeding do you remember being instructed on (you can select more than one option)?

a) Benefits of breast milk for the baby

b) Benefits of breastfeeding for the mother

c) How to position the baby and latch correctly for breastfeeding

d) Extraction, storage and freezing of breast milk

e) Donation of breast milk to human milk banks

f) Weaning

17. Until how many months of age was your baby exclusively breastfed, that is, did he not consume any other type of milk, nor liquids such as tea, juice, or any type of baby food?

18. In the last 24 hours, have you breastfed your baby at any time?

a) Yes b) No

If you answered YES:

19a. How old do you intend to continue breastfeeding your baby?

20a. What factors contributed to you continuing to breastfeed your baby up to now (you can select more than one option)?

a) Work-related issues (flexible hours, working hours, location, etc. )

b) Support from family/friends

c) Support and information obtained from health professionals

d) Knowledge about the benefits of breastfeeding

e) Personal desire

If you answered NO:

19b. At what age did you stop breastfeeding your baby?

20b. What factors contributed to you stopping breastfeeding your baby (you can select more than one option)?

a) Return to work

b) Pregnancy or birth of a younger child

c) Fatigue, exhaustion from breastfeeding

d) Difficulties with breastfeeding, such as pain, fissures, low milk production or other complications

e) Illnesses or use of medication by the mother

f) Illness or hospitalization of the baby, or difficulty gaining weight

g) Maternal/family option or preference for weaning

h) Lack of support from family/friends/health professionals

i) Others

22. Below, you will see some statements about the benefits of breastfeeding for mothers and babies. Please indicate whether you agree or disagree with these statements.

If a mother breastfeeds her baby, it reduces her risk of breast cancer.

If a mother breastfeeds her baby, it reduces her risk of type 2 diabetes.

If a mother breastfeeds her baby, it decreases her risk of having high blood pressure.

If a mother breastfeeds her baby, it decreases the risk of the baby having diarrhea.

If a mother breastfeeds her baby, it decreases the baby 's risk of respiratory diseases.

If a mother breastfeeds her baby, it reduces the risk of the baby becoming obese.

a) I strongly agree

b) I partially agree

c) I neither agree nor disagree

d) I partially disagree

e) I strongly disagree

23. Are you aware of the recommendations from the World Health Organization and the Ministry of Health regarding how long a baby should be breastfed?

"The World Health Organization (WHO) recommends that babies be exclusively breastfed until they are 6 months old. And that, even after the introduction of their first solid foods, they continue to be breastfed until at least 2 years of age."

a) yes

b) No
